# Phyto-Capped Ag Nanoparticles: Green Synthesis, Characterization, and Catalytic and Antioxidant Activities

**DOI:** 10.3390/nano12030373

**Published:** 2022-01-24

**Authors:** Mohamed G. M. Kordy, Mohammed Abdel-Gabbar, Hanan A. Soliman, Ghadah Aljohani, Mohammad BinSabt, Inas A. Ahmed, Mohamed Shaban

**Affiliations:** 1 Biochemistry Department, Faculty of Science, Beni-Suef University, Beni-Suef 62521, Egypt; m.kordybio@science.bsu.edu.eg (M.G.M.K.); hhmgabar@science.bsu.edu.eg (M.A.-G.); hanan_abdelhameid@yahoo.com (H.A.S.); 2 Nanophotonics and Applications (NPA) Lab, Physics Department, Faculty of Science, Beni-Suef University, Beni-Suef 62514, Egypt; 3 Chemistry Department, College of Science, Taibah University, Al-Madinah Al-Munawwarah 14177, Saudi Arabia; gjohani@taibahu.edu.sa; 4 Chemistry Department, Faculty of Science, Kuwait University, P.O. Box 5969, Safat 13060, Kuwait; Mohammad.binsabt@ku.edu.kw; 5 Department of Chemistry, Faculty of Science, King Khalid University, Abha 62224, Saudi Arabia; eaahmed@kku.edu.sa; 6 Department of Physics, Faculty of Science, Islamic University of Madinah, Al-Madinah Al-Munawwarah 42351, Saudi Arabia

**Keywords:** Ag nanoparticles, plasmonic nanoparticles, DPPH free radicals, dye degradation, green synthesis, wastewater treatment

## Abstract

Using a simple approach, silver nanoparticles (Ag NPs) were synthesized from green coffee bean extract. The optical color change from yellowish to reddish-brown of the green-produced Ag NPs was initially observed, which was confirmed by the UV-Visible spectrophotometer’s surface plasmonic resonance (SPR) bands at 329 and 425 nm. The functional groups of green coffee-capped Ag NPs (GC-capped Ag NPs) were studied using a Fourier transform infrared spectrometer, revealing that Ag NPs had been capped by phytochemicals, resulting in excellent stability, and preventing nanoparticle aggregation. The presence of elemental silver is confirmed by energy dispersive X-ray analysis. In addition to the measurement of the zeta potential of the prepared GC-capped Ag NPs, the size distribution is evaluated by the dynamic light scattering. Depending on the nano-morphological study, the particle diameter of Ag NPs is 8.6 ± 3.5 nm, while the particle size of GC-capped Ag NPs is 29.9 ± 4.3 nm, implying the presence of well-dispersed nanospheres with an average capsulation layer of thickness 10.7 nm. The phyto-capped Ag NPs were found to be crystalline, having a face-centered cubic (FCC) lattice structure and Ag crystallite size of ~7.2 nm, according to the XRD crystallographic analysis. The catalytic performance of phyto-capped Ag NPs in the removal of methylene blue dye by sodium borohydride (NaBH_4_) was investigated for 12 min to reach a degradation efficiency of approximately 96%. The scavenging activities of 2,2-Diphenyl-1-picrylhydrazyl (DPPH) free radicals are also examined in comparison to previously reported Ag-based nano-catalysts, demonstrating a remarkable IC_50_ of 26.88 µg/mL, which is the first time it has been recorded.

## 1. Introduction

Water pollution, in the form of hazardous contaminants that poison industrialization’s water supplies, is the most serious threat to our lives. Our main concern is a long-term water shortage caused by substantial contaminants introduced into water sources; these contaminants include a variety of organic and inorganic dyes used as colorants in the pharmaceutical, food, cosmetics, textiles, plastics, paper, ink, photographic, and paint industries [1]. Metal nanoparticle research has become an integral aspect of nanotechnology in recent years due to rapid progress. Relative to their bulk counterparts, nanoparticles with diameters of 1–100 nm possess distinctive physical/chemical properties, which can be related to their enormous surface/volume ratios and electronic features [2]. Metal nanoparticles and their oxides are employed in catalysis, biological tagging, photonics, optoelectronics, medicinal research, and the food industry for these reasons [3,4,5,6]. Silver was chosen among several metal nanoparticles for this study because of its well-known catalytic activity [7]. To synthesize silver nanoparticles, scientists can use a variety of techniques, including chemical, radiation, Langmuir–Blodgett, photochemical, electrochemical, and biological methods [6,8,9,10]. The green technique is a biochemical process that uses natural reducing agents such as live organisms or their products, such as plant extract, bacteria, fungus, or algae, to reduce metal ions for nanoparticle assembly in their own way [11]. This biological technique is a bottom-up method that assembles metallic nanoparticles from smaller entities by joining together and forming the final particles. In the beginning, the nanostructured building blocks of the nanoparticles are created, for example, by reducing a salt containing the required metal ion. After that, stabilization and capping techniques are used to keep the final particles together [12,13]. Such techniques are cost-effective and ecologically friendly. They are also known as green synthesis because they are environmentally beneficial. Plant extract phytochemicals are utilized as a reducing and capping agent in this process [14]. Capping agents are included in nanoparticles and are used to promote nanoparticle stabilization [3]. According to Rosarin et al., high-surface-area metal nanoparticles are used in drug administration with greater dosages, such as the antiproliferative action of silver nanoparticles on Hep2 cell line, manufactured using amla [3]. Ag NPs’ antibacterial activity is influenced by their size and shape, according to Ashour et al. and Nedelcu et al. [7,15]. The most effective antibacterial Ag NPs were the triangular ones mentioned by Nedelcu et al. [15]. In comparison, the smaller size of the spherical Ag NPs was likewise the ideal size for showing good antimicrobial capabilities [15]. As a result of the generation of reactive oxygen species (ROS), the bacteria can be killed or damaged [10]. On the other hand, Ag NPs were described as electrophile generator during their interaction with microorganisms due to Ag^+^ ions release. Ag^+^ ions are most likely discharged as a result of the surface interactions. This causes the microorganism to be seriously affected and damaged. The Ag^+^ ions may interact to damage the microorganisms’ negatively charged cell walls, deactivating cellular enzymes and disrupting membrane permeability, resulting in cell lysis and death [10]. The toxicity of Ag NPs to aquatic organisms is very high. Ag NPs with diameters less than 16 nm can enter the cells of various types of bacteria and the direct contact with bacterial membranes toxifies the bacteria. Different shapes, sizes, and kinds of nanoparticles, including plasmonic and metal oxide nanoparticles, were created using diverse plant extracts [16,17,18]. According to IR spectroscopic investigations, the primary phytochemicals responsible for turning silver ions into metallic Ag NPs are alkaloids, terpenoids, glycosides, and phenolics (flavonoids, quinones, coumarins, tannins, etc.) [19,20]. As a result, chlorogenic acids are the most common form of phenolic compounds produced via esterification of trans-cinnamic acids (such as ferulic, caffeic, and p-coumaric acids) with quininic acid and melanoidins [21]. These compounds are significant because of their antioxidant and antibacterial effects [22,23]. A plant extract was utilized in our investigation because biological molecules such as glycoprotein, protein, fatty acid, lipid, flavonoid, phenol, and sugar may play a role in the control of free radical generation [24]. Free radicals are generated through reactive oxygen species (ROS) such as superoxide ion, hydroxyl, hydrogen peroxides, peroxyl, singlet oxygen, ozone, hypochlorous acid, reactive nitrogen species (RNS) such as nitric oxide, peroxynitrite, and other reactive nitrogen species such as nitrosonium cation [25]. According to Makarov et al., flavonoids can bind and actively reduce metal ions into metallic nanoparticles because they contain several hydroxyl and carbonyl groups [26]. In prior research, Ag NPs made from aqueous extracts of garlic, ginger, and cayenne pepper were found to have a significant scavenging activity for 2,2-diphenyl-1-picrylhydrazyl free radicals [27]. Several researchers have established the methods of silver nanoparticle creation using various biological sources, as well as its use in eliminating colors from water. Metallic nanoparticles are thought to be promising due to their antibacterial capabilities, which are attributable to their high surface-to-area ratio. Green synthesis is a cost-effective, environmentally zero-energy-based, friendly, and less time-consuming technique that does not employ any harmful chemicals. Moreover, it does not require the use of any kind of stabilizers. Under green synthesis, a variety of environmentally acceptable components such as bacteria, fungi, algae, plant extracts, and enzymes are categorized and used as sources [28]. According to Tagad et al., Ag NPs manufactured using a green approach are extremely compatible for pharmaceutical and other bio-medical applications, including bio-sensing for hydrogen peroxide, which is harmful and causes free radicals inside living cells [29]. This method may simply be scaled up for bulk Ag NP synthesis without the utilization of high pressure, energy, or temperature conditions [30]. *Coffea arabica* (family; Rubiaceae) beans are one of the most frequent plant products consumed on a regular basis. The extract of Arabic green coffee beans was used as a plant extract for reduction and stabilization functions in this experiment. It is a popular hot beverage in the Middle East, particularly in Saudi Arabia, and it is well known for its many uses. Arabica green coffee extract contains polyphenols such as caffeine, according to phytochemical studies [13]. Terpenoids, alkaloids, glycosides, and phenolic compounds (flavonoids, coumarins, quinones, tannins, etc.) are discovered to be responsible for the reduction of silver ions into silver nanoparticles and for their stability, as observed in IR spectra in previous research [19,20]. This plant’s bean was previously discovered to possess high quantities of phenolic chemicals, suggesting that it could be employed as a bio-reductant to create Ag NPs [19,20]. According to Wang et al., green coffee is employed in the green production of copper nanoparticles to catalyze the reduction of organic dyes such as amido black 10B, methylene blue, and xylol orange [31].

The fabrication of silver nanoparticles utilizing phytochemicals entails three processes, as previously stated. Firstly, the Ag atoms are formed through the reduction of Ag^+^ by bio-reductants, which are reducing agents of GC extract. The Ag atoms nucleate to develop Ag NPs; secondly, the Ag NPs grow to larger ones after the reduction of Ag^+^ ions. That results in the formation of Ag NPs that need to be stabilized. Thirdly, electrostatic stabilizing agents can control the size of the Ag NPs through the capping process [32]. The capping process requires the stabilizers to be adsorbed onto the surface of Ag NPs to be capped. The capping agents are also the GC extract in our study. This occurred by the adsorption of excessive negatively charged reducing agent ions on the surface of the formed Ag NPs [33]. Over the years, several plant extracts have been utilized as reducing agents in the biological manufacture of Ag NPs. One of the earliest works of plant-mediated bio-synthesis of Ag NPs was carried out by Shankar et al. with Geranium leaf extract as a reducing agent [34]. Using the fruit extracts of *Emblica Officinalis*, Ankamwar et al. showed a quick reduction and stable production of Ag NPs [35]. Chandran et al. employed ethanolic or aqueous aloe vera plant extract as a bio-reductant to manufacture Ag NPs in the employment of mercury II removal [16]. They asserted the probable reason for the spherical shape of the synthesized Ag NPs. The callus extract was also used by Mude et al. for Ag NPs synthesis, demonstrating the rapid synthesis of Ag NPs in the range of 60–80 nm [36]. Another study of the bio-reduction of Ag NPs by the fruit extracts of Indian gooseberry (*Phyllanthus emblica*) was carried out by Renuka et al. [37]. They reported a short formation time concerning previous research works. A significant antimicrobial effect was reported in their research work.

Every year, industries utilize a huge number of dyes and pigments. The development of analytical techniques to eliminate these contaminants from wastewater is critical since these hazardous chemicals may create severe issues for human health and the environment. NPs generated from biogenic sources have been reported as a simple and cost-effective way to catalyze the removal of pollutants in this sector. This synthesis is useful in treating free radicals and synthetic dyes, which are mainly present in wastewater and cause pollution to our environment. So, metal nanoparticles such as Ag nanoparticles can incorporate the treatment of these pollutants from wastewater through the antioxidant and catalytic activities of these metallic nanoparticles. Methylene blue dye through a reduction reaction by sodium borohydride is a catalyst because it is distinguished by its excellent catalytic activity. In this present study, the aqueous extract of green coffee beans has been used for the investigation of the biosynthesis of Ag NPs and to show their activities as antioxidants and as catalysts for methylene blue dye reduction by sodium borohydride. In this study, we prepared silver nanoparticles using green coffee bean extract as a reducing and capping agent, as reported by Wang et al. for first-time preparation, with some modifications [38]. Biosynthesis of Ag NPs using plants or microorganisms is a well-known approach for the development of safe and competent control strategies against resistant bacteria [28].

GC beans extracts were obtained from the seeds of *Coffea Arabica*. They have been used as a healthy food supplement for a long time because the main ingredient is chlorogenic acid, which reduces relative blood pressure, in addition to their native antioxidants capability [39]. We used SEM, EDX, FTIR, TEM, DLS, zeta potential, and XRD for characterization of the biosynthesized Ag NPs using GC extract. The formation of Ag NPs was recorded by a UV-Visible spectrophotometer. The as-prepared Ag NPs sample was used as the catalyst for the reduction of MB dye in presence of NaBH_4_, which was kinetically evaluated in the expression of rate constant and catalytic activity. Moreover, the antioxidant ability of these nanoparticles to scavenge DPPH free radicals is also studied.

## 2. Experimental Details

### 2.1. Materials

Unroasted dried green coffee beans were purchased from a local market, and silver nitrate (AgNO_3_) was purchased from TITAN BIOTECH (New Delhi, India) with a ≥99.5% purity for the synthesis of Ag NPs. Methylene blue (MB) dye was used as a standard dye supplied by the Al-Nasr Company (Giza, Egypt). 2,2-diphenyl-1-picrylhydrazyl (DPPH) free radicals were purchased from Himedia Laboratories (Mumbai, India) dissolved in methanol HPLC grade from Fisher (Loughborough, UK) to measure the antioxidant activity of GC-capped Ag nanoparticles. All these purchased reagents were analytical grade and used without any further purification. Double distilled water was used for preparing aqueous solutions all over the experiment.

### 2.2. Preparation of GC Beans Extract

**Figure 1:**
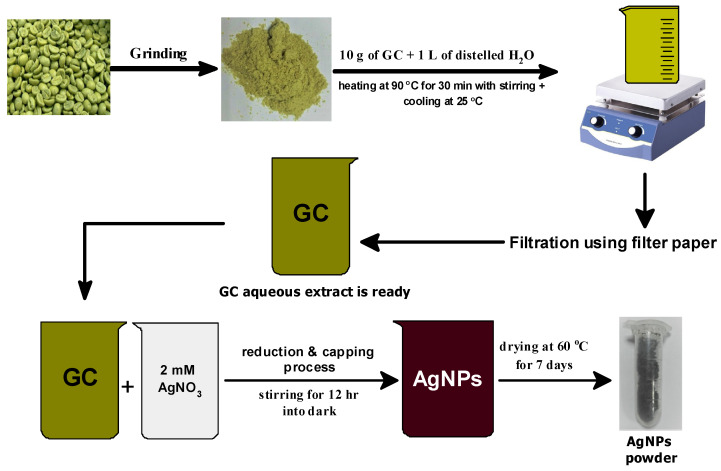
Schematic diagram of the preparation method of phyto-capped Ag NPs.

The following technique was used to make aqueous seed extract, Figure 1. Fresh seeds of unroasted green coffee beans were first cleaned, then dried and ground thoroughly. Ten grams of freshly ground green coffee was weighed and combined with 1 L of distilled water in a beaker. The mixture was heated to 90 °C for 30 min with constant mechanical stirring, then cooled to room temperature. Finally, the mixture was filtered using a filtering system, and the extract was kept at 4 °C in the refrigerator until it could be used to manufacture Ag NPs using AgNO_3_.

### 2.3. Synthesis of GC-Caped Ag Nanoparticles

Figure 1 displays the schematic diagram of the preparation method of phyto-capped Ag NPs. The 2 mM AgNO_3_ solution was made by dissolving AgNO_3_ crystals in distilled water. The AgNO_3_ solution was treated with an aqueous extract of green coffee beans in a 1:1 ratio with a total volume of 1 L in a beaker covered with aluminum foil and maintained in a dark environment for 12 h with constant stirring. The color changed from pale yellow to dark brown, indicating that Ag nanoparticles had been synthesized. The produced mixture was dried completely by incubation at 60 °C for 7 days. Because 1 L of the mixture was used, as previously stated, it took seven days to dry and allow all the water containing volatile organic compounds to be evaporated thoroughly. The sample was purified by dissolving it in 100 mL of pure water and centrifuging for 15 min at 6000 rpm. Furthermore, the nanoparticles were collected and dried at 60 °C. The well-dried sample was kept at 4 °C in the dark for future use to avoid any further thermal or photocatalytic activity.

### 2.4. Characterization of Biologically Synthesized Ag NPs

A double-beam LAMBDA 950–UV-Visible spectrophotometer (Perkin Elmer, Boston, MA, USA) was used to scan the optical characteristics for the aqueous extract of green coffee and the solution of GC-capped Ag NPs. The functional groups of chemical components were identified using Fourier transform infrared spectroscopy (FTIR-8400 S Shimadzu, Kyoto, Japan) from 4000 to 400 cm^−1^ with a resolution of 4 cm^−1^ at 25 °C, utilizing the KBr pellet technique. The size and shape of the crystalline Ag NPs were examined using a high-resolution transmission electron microscope (HRTEM) (JEOL-2010F) (Tokyo, Japan) and a size distribution histogram using Image-J software version 1.53e (Whayne Rasband and contributors, National Institute of Health, New York, NY, USA). Scanning electron microscope (SEM) (JSM-6510, JEOL) (Tokyo, Japan) micrographic images were obtained at different magnifications. To examine peaks alterations in X-ray diffraction studies, the crystallography of the phyto-capped Ag NPs and green coffee powder were detected using (XRD, Philips X’Pert Pro MRD) (Malvern, UK). XRD charts were measured using Cu-Kα radiation (λ = 0.154056 nm) operated at 40 kV and 40 mA with a scanning rate of 0.01°/s in 2θ-range from 10 to 80°. An energy-dispersive X-ray detector (EDX, JED2300 Analysis station, JEOL, Tokyo, Japan) equipped on SEM was used to confirm the elemental composition of the samples. Finally, using the Zeta Sizer Nano-series, dynamic light scattering (DLS, Nano-ZS90, Malvern, UK) was used to determine the particle size distribution and zeta potential of the prepared phyto-capped Ag NPs.

### 2.5. Catalytic Reduction of MB by GC-Capped Ag Nanoparticles Using Sodium Borohydride

Catalytic reduction of 50 ppm MB by the as-synthesized 500 µg/mL Ag NPs suspension was assessed using 0.1 M NaBH_4_ from Research Lab Fine Chem Industries (Mumbai, India). The test samples were prepared by adding 100 µL Ag NPs to a mixture of 1 mL of MB and 1 mL of NaBH_4_. The absorbance was monitored on UV-visible spectrophotometer (Perkin Elmer 950-lambda, Boston, MA, USA) at 665 nm and scanned from 500 to 700 nm. The UV-visible spectra were also recorded with sequential time intervals of incubation set off from 0 to 12 min in dark conditions to prevent the photocatalytic reaction. If GC-capped Ag NPs are widely used, their discharge and subsequent leaching into surface waters may endanger aquatic species because AgNPs are highly toxic. As a result, after use, it is critical to remove GC-capped Ag NPs via dialysis.

The as-synthesized sample’s catalytic efficiency of MB reduction was calculated as follows; Equation (1) and the linear relationships between $\ln\left(\frac{A_{t}}{A_{o}}\right)$ and reaction time (min) can be attributed to pseudo-first-order kinetics, indicating the excessive existence of NaBH_4_ in the reaction by the Equation (2):

(1) $$\mathrm{Catalytic}\;\mathrm{reduction}\;\%=\frac{A_{o}-A_{t}}{A_{o}}\times 100$$

(2) $$\ln\left(\frac{A_{t}}{A_{o}}\right)=-k_{app}t$$

where *A_t_* and *A_o_* are the absorbances of MB at time *t* and zero, respectively. The reaction rate constant (*k_app_*) was determined from the linear plot of $\ln\left(\frac{A_{t}}{A_{o}}\right)$ against time (min) ranges from 0 to 12 min.

### 2.6. Antioxidant Activity Test of Green Synthesized GC-Capped Ag NPs against DPPH Free Radical

The antioxidant activity of the GC-capped nanoparticles was determined using the DPPH method according to Ali et al. [40] and Cuvelier et al. [41], with some modifications, and 1 mL of 100 µM methanolic solution of DPPH, and 3.94 mg of DPPH dissolved in 100 mL methanol, was added to 1 mL of GC-capped Ag NPs dissolved in deionized water at doubled concentrations (3.125, 6.25, 12.5, 25, and 50 µg/mL). The colorimetric absorbance measurements were performed at 517 nm after 30 min in triplicate to get the average value. The scavenging activity of GC-capped Ag NPs is expressed by inhibition % of DPPH free radicals and calculated by determining the decrease in absorbance by the successive addition of GC-capped Ag NPs. The activity to scavenge DPPH radicals was estimated by Equation (3):

(3) $$Scavenging\;Activity\;\%=\left[1-\frac{A_{s}}{A_{C}}\right]\times 100$$

The absorbances of the control and the test samples are represented by $A_{C}$ and $A_{s}$, respectively. The control of DPPH was generated without the inclusion of any GC-capped Ag NPs.

## 3. Results and Discussion

### 3.1. Characterization of Biologically Synthesized Ag NPs

**Figure 2:**
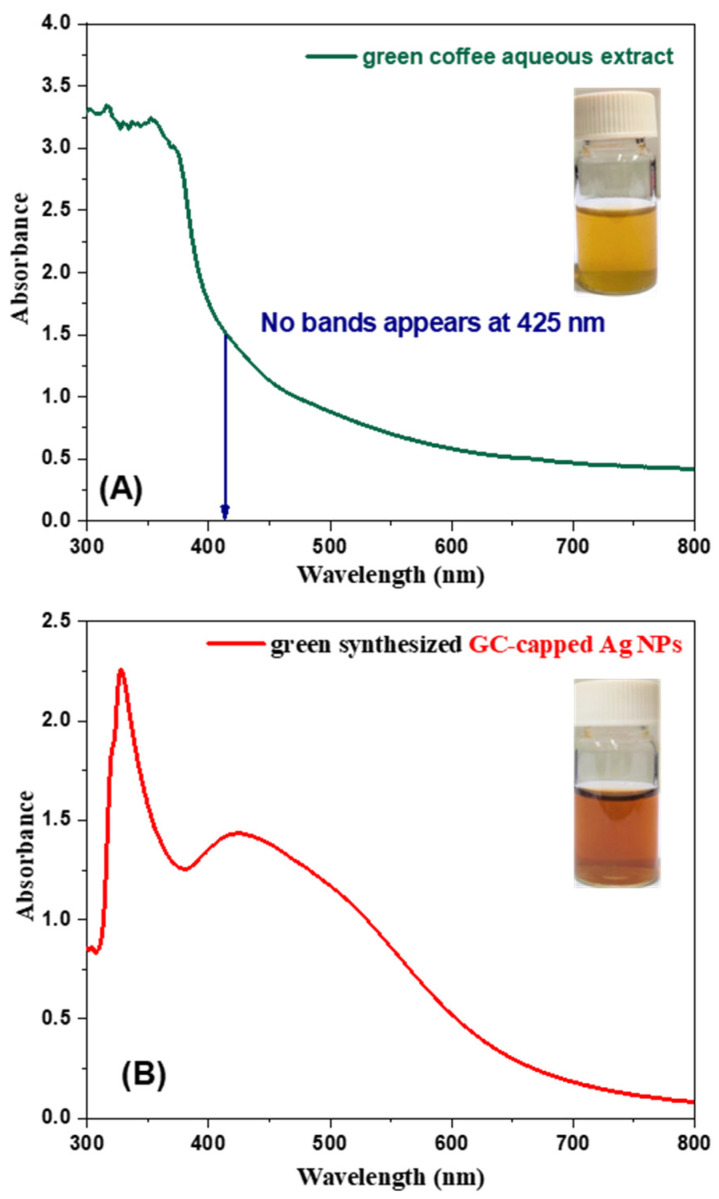
Absorbance spectra of (**A**) aqueous extract of green coffee and (**B**) GC-capped Ag NPs. Insets show photographs of the extract (**A**) and suspension of GC-capped Ag NPs in distilled water (**B**).

The reduction of Ag^+^ was confirmed by comparing the reaction mixture’s UV-Vis spectra to distilled water as a blank. UV/Vis spectral analysis was performed using a double beam Perkin Elmer spectrophotometer with a resolution of 1 nm from 300 to 800 nm for the aqueous extract of green coffee and GC-capped Ag NPs, Figure 2. The pale yellow color of the aqueous extract with no evident bands, Figure 2A, changes to dark brownish color with discernible bands at 329 and 425 nm, Figure 2B, suggesting the formation of capped-silver nanoparticles. The surface plasmon resonance (SPR) of GC-capped Ag NPs was detected at 425 nm, confirming the reduction of pure silver ions and capping of the Ag NPs [42]. As the GC-capped sample can be considered to some extent to monodisperse with spherical NPs, the UV peak at 329 nm may be ascribed to the GC shell of Ag NPs or some other charge transfer band. Looking at the SPR peak from 425 nm, one can see the asymmetrical shape of the broad band, i.e., the shoulder of the SPR peak towards 500 nm being from the aggregation. In other words, our natural organic capped Ag NPs had a higher primary absorption peak intensity, implying a larger population of monodisperse particles and slower aggregation formation in the event of secondary absorption peak, which is consistent with Gunsolus et al. [42]. Green coffee extract was employed as a reducing and stabilizing agent at the same time, similar to how soap-root plant extracts are used as stabilizers and manna of hedysarum plant extracts are used as reducers in the production of organic-capped Ag NPs [43].

**Figure 3:**
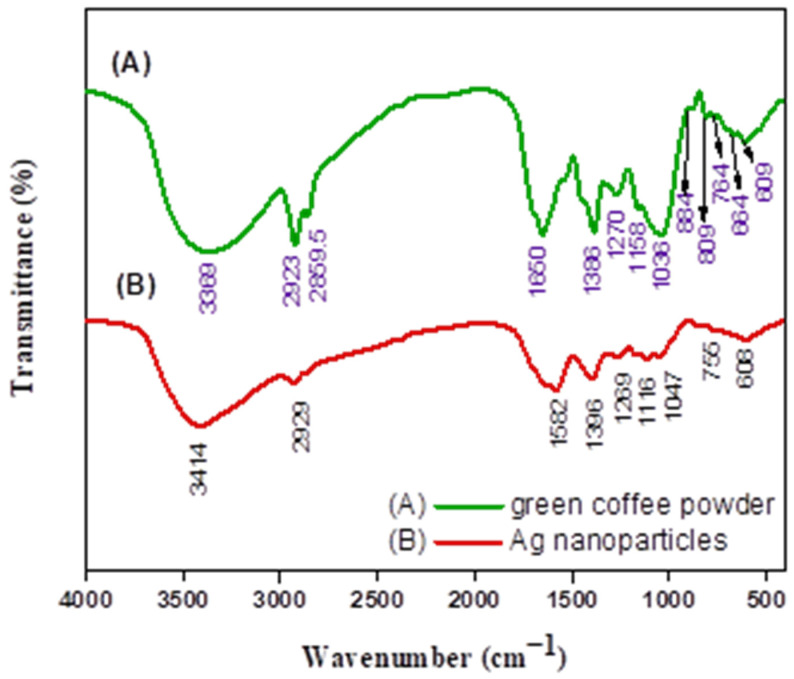
FTIR spectra of green coffee powder (**A**) and GC-capped Ag NPs (**B**).

Figure 3 shows the potential biomolecules present in the GC extract that are responsible for the reduction of silver ions and their interaction with the produced Ag NPs for stabilization. Peaks at 3369, 2923, 2860, 1650, 1386, 1270, 1158, 1036, 884, 809, 764, 664, and 609 cm^−1^ were found in the FTIR spectrum of green coffee powder, Figure 3A. The FTIR spectrum of Ag NPs, Figure 3B, displays intense bands at 3414, 2929, 1582, 1396, 1269, 1116, 1047, 755, and 608 cm^−1^. Additionally, significant differences were observed between the spectral positions of FTIR bands in the powdered green coffee and GC-capped Ag NPs due to the reduction and stabilization process. The broadband at 3414 cm^−1^ in Figure 3B represents the strong stretching vibrations of the hydroxyl group (-OH) of phenolic compounds, flavonoids, and triterpenoids; this broadband was displaced from green coffee’s 3369 cm^−1^ in Figure 3A. As noted by Farah et al., this crucial shift indicates the participation of polyphenolic compounds in the reduction process [44]. The C–H stretching bands may be ascribed to the sharp two strong peaks at 2923 and 2860 cm^−1^ in Figure 3A, indicating the existence of aliphatic compounds in the powdered green coffee that may have contributed to the reduction process [45]. The FTIR of green coffee exhibits a strong band at 1650 cm^−1^ corresponding to C=O (amide I) stretching mode. This peak shifted to 1582 cm^−1^ in Figure 3B, suggesting the possible association of the above-mentioned group in Ag NPs synthesis. The amide I band indicates that proteins can bind to Ag^+^ through carboxylate ions or free amine groups. In addition, the medium band at 1386 cm^−1^ refers to O-H bending of phenol or presence of C-H group that increased to 1396 cm^−1^ in GC-capped Ag NPs spectrum. The FTIR bands at 1270, 1158, and 1036 cm^−1^ can be attributed to C-O stretching vibrations of carboxylic acid, ester, or ether groups of the proteins or the presence of polysaccharides in green coffee. These peaks are also shifted to 1269, 1116, and 1047 cm^−1^ in the GC-capped Ag NPs spectrum. The fingerprint region from 1000 to 400 cm^−1^ has five peaks in the case of green coffee powder and two peaks only for Ag NPs. Thus, based on the FTIR spectra, it can be assumed that these natural organic compounds play a major role in both the reduction of Ag^+^ and the stabilization of the green-produced Ag NPs. According to Li et al., polyphenolic substances such as rosmarinic acid, caffeic acid, protocatechuic acid, chlorogenic acid, vanillic acid, ferulic acid, gallic acid, apigenin, and luteolin aid in the bio-reduction process [46].

**Figure 4:**
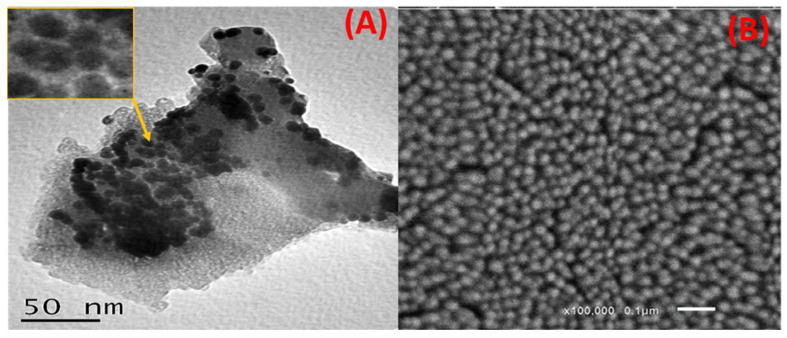
**(A)** HRTEM and (**B**) SEM images of the capped Ag NPs.

**Figure 5:**
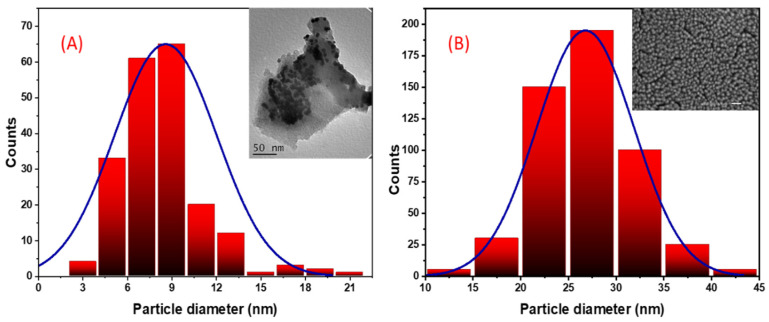
(**A**) Diameter distribution histograms and HRTEM image (insertion) of Ag NPs; (**B**) Diameter distribution histograms and SEM image (insertion) of GC-capped Ag NPs.

The size and morphology of GC-capped Ag NPs synthesized using an aqueous extract of green coffee have been evaluated by TEM and SEM analysis, Figure 4. The nanoparticles are quite poly-dispersed, with spherical or semispherical nanomorphologies, in addition to the organic layer that encapsulates the generated Ag NPs as illustrated in Figure 4A. This could explain the nanoparticles’ good dispersion in the solution, which was used as a reducing and capping agent at the same time [47,48]. As a result, the Ag nanoparticles generated using aqueous extracts of green coffee are well distributed, notwithstanding some minor agglomerations seen in Figure 4A. The presence of various polyphenolic components, such as flavonoids and terpenoids, aided the reduction of Ag^+^ and stabilized the surface of the capped-Ag nanoparticles [48]. Using the image-J program, the diameters of the Ag nanospheres are obtained from Figure 4A. Figure 5A depicts a histogram of the particle diameter distribution. The average diameter of the particle is estimated to be 8.6 ± 3.5 nm.

Figure 4B shows SEM image of phyto-capped Ag NPs homogenously distributed over the carbon tap. Almost all capped particles are of spherical nanomorphology with uniform size. No evidence of agglomeration is observed in this image, which implies again the role of polyphenolic components in the reduction of Ag^+^ and stabilized the surface of the GC-capped Ag NPs. This also confirms the strong surface plasmons of the capped Ag NPs observed in the absorption spectra of Figure 2B. Figure 5B depicts a histogram of the GC-capped Ag NPs diameter distribution. The average GC-capped Ag NPs diameter is estimated to be 29.9 ± 4.3 nm. This means that the average thickness of the capsulation layer is ~10.7 nm.

**Figure 6:**
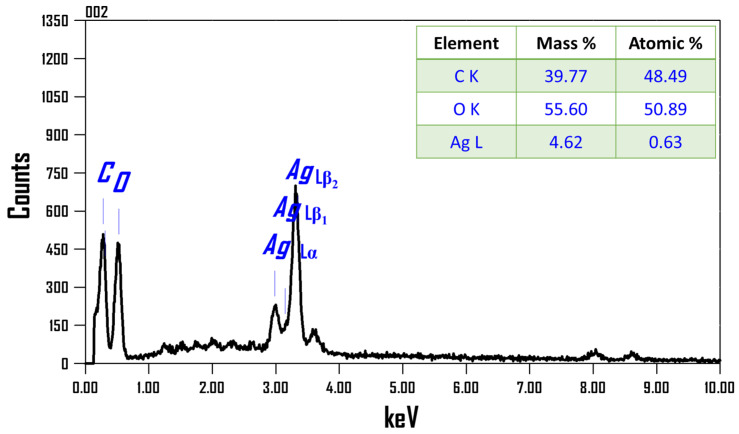
EDX pattern of the GC-capped Ag NPs with their elemental composition percentages.

The prepared GC-capped Ag NPs sample was investigated using an EDX-SEM (JED-2300 Analysis station) at 30 kV and 33X magnification with a probe current of 1.0 nA to determine the presence of elemental silver and its percentage, to confirm the reduction process of Ag^+^ into Ag. The EDX spectrum, which ranges from 0 to 10 keV, illustrated the presence of obvious Ag signals at 2.983 keV, Figure 6. This confirms its elemental constituents with a low mass percentage of 4.62%; this minor proportion is due to using a very low concentration of silver nitrate solution regarding other elements that represent the all-sample matrix. The all-sample matrix utilized in the green synthesis is the organic extract, GC extract, which includes carbon and oxygen at percentages of 39.77 and 55.60%, respectively. This analysis of research goal is to synthesize Ag NPs using the smallest number of ingredients possible, such as silver nitrate, to produce fewer toxic metallic organic nanoparticles that can scavenge DPPH free radicals present in wastewater.

**Figure 7:**
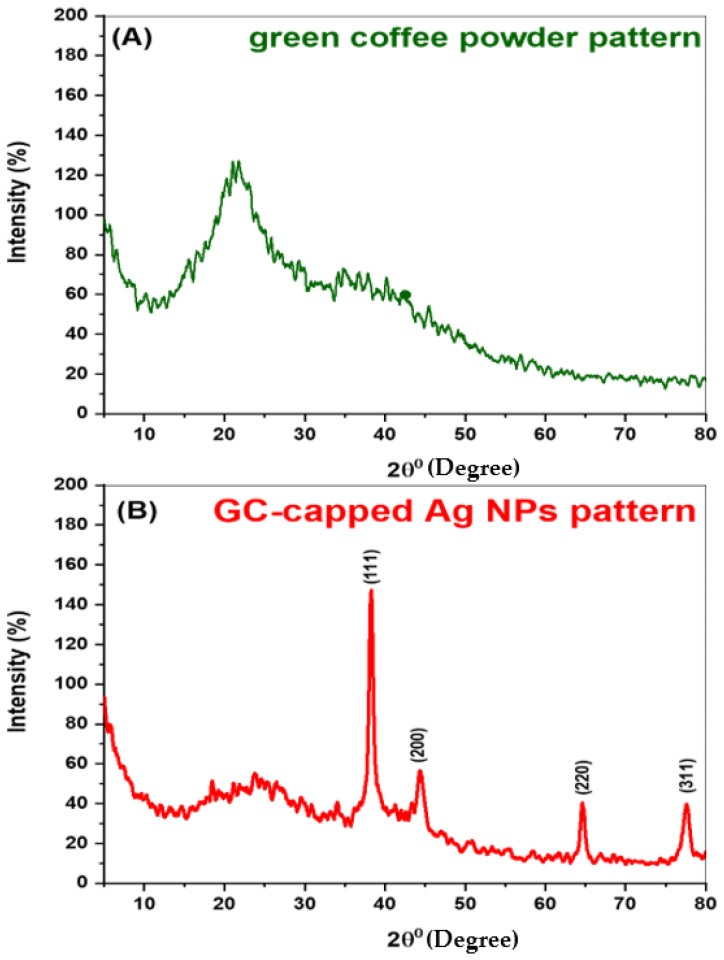
XRD patterns of green coffee beans powder (**A**) and Ag NPs (**B**).

The XRD patterns of the green coffee powder and GC-capped Ag NPs were obtained using a Philips X’Pert Pro MRD. The crystallinity nature and phase composition of metallic nanoparticles are key factors affecting their proper catalytic and biological functions. The formation of single-phase crystalline silver is the main purpose to determine the development of elemental silver. Figure 7 shows two XRD patterns of green coffee powder (A) and GC-capped Ag NPs (B). The GC-capped Ag NPs shows XRD pattern with strong and intense main diffraction peaks at 38.31, 44.42, 64.67, and 77.54°, respectively, indexed to the (111), (200), (220), and (311) diffraction planes. The diffraction peaks and planes indicated the crystalline nature of GC-capped Ag NPs with a face-centered cubic (FCC) lattice structure, which agrees with metallic silver nanoparticles (JCPDS File No. 89-3722). There was no phase other than FCC form in the GC-capped Ag NPs. The crystallite size in nm (D) was determined using the Debye–Scherer Equation (4):

(4) $$D=\frac{K\lambda }{\beta_{0}\;cos\theta }$$

where *K*, *λ*, *β*_0_, and *θ* are Scherer constant (0.95), X-ray wavelength (0.154 nm), FWHM (full width at half maximum), and Bragg’s angle in radians, respectively. The average crystallite size using the assigned four peaks in Figure 7B was ~7.2 nm. Our XRD pattern is consistent with those of previous studies [46,47,48]. Ag NPs synthesized using *Tithonia diversifol* [49], *Artemisia turcomanica* [50], and *Ziziphus Jujuba* [51] are all nanocrystals with FCC structure. Moreover, at 2θ ranges from 10 to 30°, a broad peak was observed in the two patterns in Figure 7, indicating the amorphous phase of the several organic compounds in the green coffee extract. This peak in diffractogram (B) shifted to a higher 2θ range relative to that in the diffractogram (A), perhaps due to bonding between the organic compounds that exist in the extract and metallic Ag NPs and due to the chemical changes between Ag^+^ and the green coffee extract. The observations from the XRD analysis can be correlated with the values obtained from TEM/SEM images. Because of the good agreement between XRD crystallite size and TEM nanoparticle size, the sample can be considered as being composed of single crystal silver nanoparticles.

**Figure 8:**
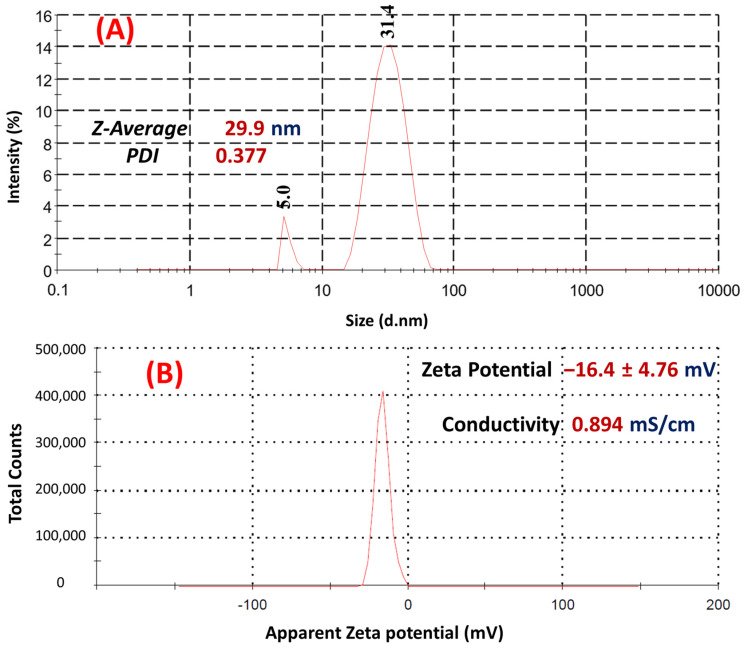
DLS (**A**) and Zeta potential (**B**) of GC-capped Ag NPs.

In colloidal solutions, DLS was used to analyze quantitative size distributions and a more accurate number of monodispersed particles. Figure 8A indicates that at two separate peak positions, the average particle size was 5.0 and 31.4 nm, with intensity percentages of 5.5 and 94.5%, respectively. With a polydispersity index (PDI) of 0.377, the auto-calculated Z-average was 29.9 nm. At 25 °C, the zeta potential (ZP) was determined to be −16.4 mV, Figure 8B, indicating the potential stability of the colloidal solution. Phyto-synthesized Ag NPs with *Pterodon emarginatus* [52], *Juglans regia* [53], and *Punica granatum* [54] had PDI values of 0.372, 0.4, and 0.54, and ZP values of −23.9, −33.8, and −34.3 mV, respectively. The DLS results support the polydispersity and long-term stability of phyto-synthesized green coffee Ag NPs. The Z-average of the particles was very close to the data obtained by SEM results.

### 3.2. Catalytic Reduction of MB by Ag Nanoparticles Using Sodium Borohydride

**Figure 9:**
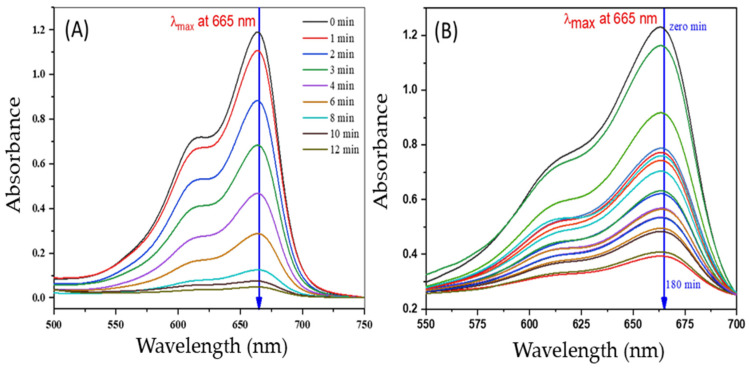
UV-Vis absorbance spectra of (**A**) Ag NPs in a mixture liquor containing NaBH_4_ and MB, and (**B**) the same mixture without Ag NPs at different reaction times.

**Scheme 1:**
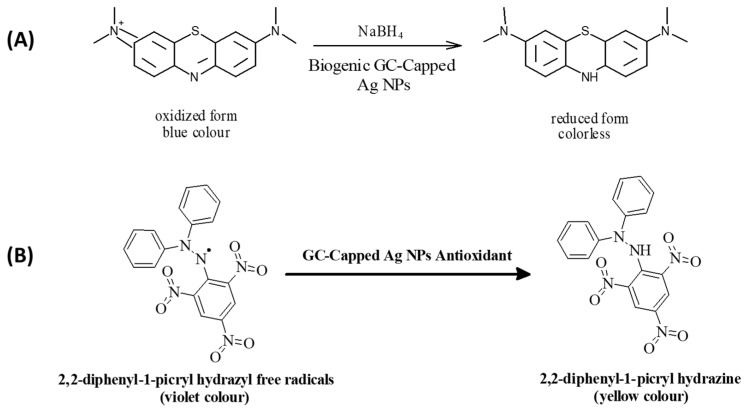
(**A**) Catalytic reduction of MB dye and (**B**) reduction of DPPH using GC-capped Ag NPs.

**Figure 10:**
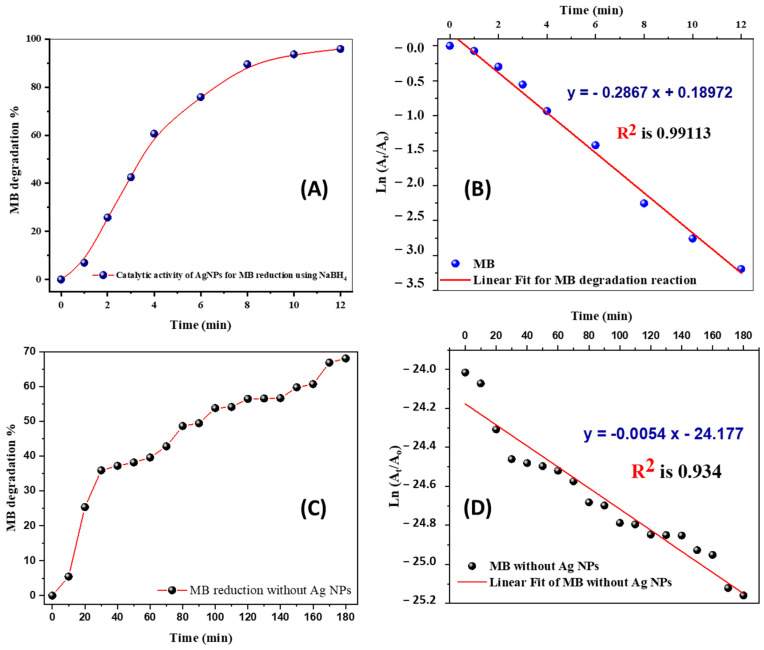
The catalytic reduction percentage (%) of MB versus the reaction time in the (**A**) existence and (**C**) absence of the GC-capped Ag NPs catalyst; (**B**,**D**) are pseudo-first order kinetics; the linear relationship between $\ln\left(\frac{A_{t}}{A_{o}}\right)$ and time (min) is shown above.

A potential application of GC-capped Ag NPs catalytic activity was the reduction of aqueous MB to Leuco-MB in the presence of excess NaBH_4_. The reaction was monitored by a UV-Vis spectrophotometer in the wavelength range between 500 and 750 nm at room temperature. In an aqueous medium, MB shows the maximum absorption peaks at 665 nm [55]. Reddy et al. mentioned that the reduction of MB by NaBH_4_ in the absence of nano-catalyst was recorded for a period of 120 min with a small decreasing trend of the maximum absorbance indicating the reduction of MB at a very slow rate [56]. The UV-Vis spectrum of the reduction of MB by NaBH_4_ in the presence of catalytically active GC-capped Ag NPs is shown in Figure 9. The reduction process was found to be accelerated in the presence of GC-capped Ag NPs colloids, which showed a rapid decrease in the absorption intensity of MB solution. It is thought that GC-capped Ag NPs help in the electron relay from BH_4_^−^ (donor) to MB (acceptor). BH_4_^−^ ions are nucleophilic, while MB (cationic dye) are electrophilic in nature with respect to GC-capped Ag NPs, where the Ag NPs accept electrons from BH_4_^−^ ions and deliver them to the MB, as shown in Scheme 1A [57]. The absorption peak at 665 nm for MB dye was found to decrease only gradually with the increase in the reaction time, indicating that the dye has been degraded at a faster rate than those reported previously without adding nano-suspension catalyst [56,58]. The degradation efficiency was calculated using Equation (1) and presented in Figure 10A. These GC-capped Ag NPs exhibit high catalytic degradation efficiency of approximately 96% after 12 min of 50 ppm of MB dye using 0.1 M of NaBH_4_. The linear correlation between $\ln\left(\frac{A_{t}}{A_{o}}\right)$ versus reduction time in minutes, Figure 10B, indicates that the reduction follows a pseudo-first order reaction kinetics with respect to 50 ppm MB dye and 0.1 M NaBH_4_ reductant. The rate constant is calculated from the slope of this graph and is found to be 0.2867 min^−1^. The GC-capped Ag NPs synthesized from green coffee clearly operate as an efficient catalyst for the breakdown of methylene blue, based on these findings and according to Table 1 [59,60,61,62,63,64]. This table showed that our capped Ag NPs are faster than most of the previously reported Ag-based nano-catalyst regarding reduction efficiency and rate constant [59,60,61,62,63,64]. In addition to the high *k_app_* value and rapid reaction, our produced capped Ag NPs is a very efficient catalyst compared to previously reported Ag-based nanocatalysts since we employed a very small dose of our nano-catalyst to degrade a very high concentration of MB (50 ppm) [59,60,61,62,63,64]. The efficiency of MB reduction without the use of our catalyst, Figure 10C, reached approximately 68% after 180 min. In the absence of the capped Ag NPs catalyst, Figure 10D, the *k_app_* was estimated to be 0.0054 min^−1^, which is extremely low when compared to its value in the presence of GC-capped Ag NPs catalyst. Using a simple mathematical calculation, $\frac{{\left(K_{app}\right)}_{with\;catalyst}}{{\left(K_{app}\right)}_{without\;catalyst}}$, our catalyst boosted the rate of MB reduction by ≈53 times. Because of their small size, large surface area, and single crystal structure, the GC-capped Ag NPs significantly increased the rate of MB reduction. Furthermore, E° (MB) = + 0.01 V > E° GC-AgNPs (−0.0164 V) > E° (BH_4_^−^) = −0.21 V, which represents the ideal conditions for an effective relay of electrons between the acceptor (MB) and the donor (NaBH_4_). Therefore, the GC-capped AgNPs constituted the electrophiles that could take electrons from BH_4_^−^ and transport them to MB at a faster rate than the other electrophiles that are listed in Table 1. These findings show that our eco-friendly GC-capped Ag NPs catalyst has a strong capacity to efficiently remove large amounts of hazardous dyes from water in only a few minutes.

### 3.3. Antioxidant Activity Test of Green Synthesized GC-Capped Silver Nanoparticles against DPPH Free Radical

**Figure 11:**
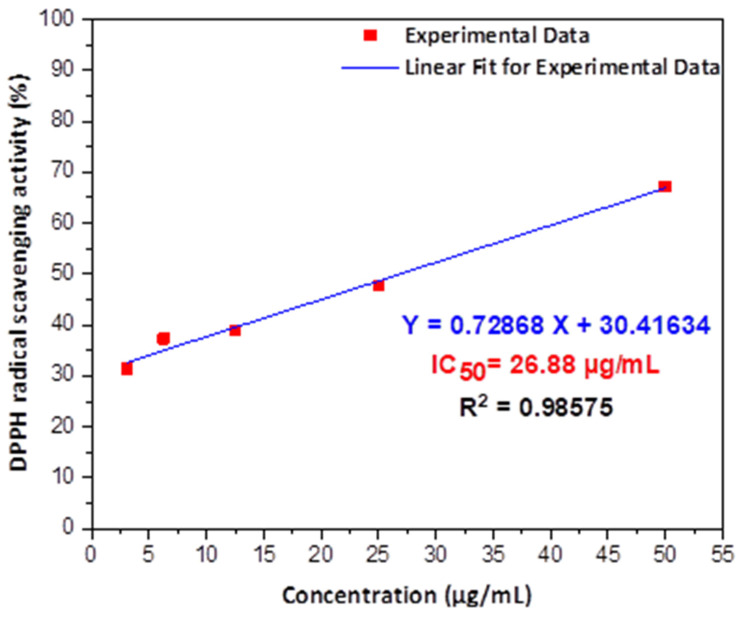
The antioxidant activity of GC-capped Ag NPs using different concentrations to determine the IC_50_ after linear fitting for experimental data.

Figure 11 shows the values of DPPH scavenging activities of GC-capped Ag NPs against their concentrations in µg/mL with IC_50_ and R-square calculations. The scavenging activity of GC-capped Ag NPs was increased with increasing the concentration of prepared aqueous Ag nanoparticles. A linear fitting is applied for the direct proportionality using OriginPro 2018 software (OriginLab Corp., Northampton, MA, USA). As shown in Figure 11, we can conclude the strong ability to scavenge the free radicals of DPPH (purple color, Scheme 1B in 30 min using different concentrations of GC-capped Ag NPs (pale-yellow color, Scheme 1B with IC_50_ of 26.88 µg/mL. This is due to our Ag NPs antioxidant’s ability to donate hydrogen or transfer an electron to stable free radicals [65]. The IC_50_ refers to the concentration of antioxidants that scavenge 50% of free radicals of DPPH. The value of R^2^ is 0.98575 for validation of the fitting result. Table 2 illustrates the values of our IC_50_ relative to previously reported data for biogenically derived plasmonic nanoparticles utilizing DPPH free radicals [46,66,67,68,69]. Our IC_50_ value is more effective and lower than those obtained by Li et al. using Perilla frutescens leaf extract and Das et al. using the outer peel extract of Ananas comosus [46,66]. Furthermore, Niraimathi et al. employed *Alternanthera sessilis* (Linn.) leaf extract to fabricate Ag NPs with IC_50_ = 403.19 µg/mL, which is a higher value than ours [68]. Additionally, our IC_50_ value is lower than that obtained by biogenic CGA@Au NPs produced from Couroupita guianensis Aubl fruit extract (37 µg/mL against DPPH), according to Sathishkumar et al [70]. Our IC_50_ value is the best achievable when compared to the various IC_50_ values presented in Table 2. Furthermore, free radical scavenging materials in living organisms play an important role in adaptive immunity, the anti-aging system, and anti-carcinogenesis, according to earlier studies [46,71].

## 4. Conclusions

The GC-capped Ag NPs were synthesized from green coffee bean extract. Their surface plasmonic resonance (SPR) bands at 329 and 425 nm confirm the capped structure of crystalline silver nanoparticles with a characteristic reddish-brown color. The GC-capped Ag NPs are characterized using FTIR, EDX, DLS, zeta potential, HRTEM, and SEM. XRD revealed the face-centered cubic structure of GC-capped Ag NPs with crystallite size ~7.2 nm. These GC-capped Ag NPs exhibit high degradation efficiency of approximately 96% in catalytic reduction of 50 ppm of MB dye using 0.1 M of NaBH_4_ with k_app_ of pseudo-first-order reaction equal to 0.2867 min^−1^. This maximum catalytic activity was achieved by GC-capped Ag NPs in 12 min, which is considered the quickest reaction time in comparison to the previously reported literature. Additionally, GC-capped Ag NPs exhibit strong antioxidant activity, IC_50_ = 26.88 µg/mL, which is reported for the first time against 2,2-diphenyl-1-picrylhydrazyl (DPPH) free radicals. The achievement of synthesizing eco-friendly GC-capped Ag NPs with very small diameters and confirming their SPR at two selected peaks was the work’s originality. Their application in the treatment of contaminated water has been shown to be the fastest and the most effective against high concentrations of MB dye and DPPH free radicals relative to any previously reported Ag-based nanocatalysts.

## Figures and Tables

**Table 1:** Comparison between different biogenic silver-based nano-catalysts and our GC-capped Ag NPs concerning reaction and performance parameters.

No.	Catalyst	Source of Synthesis	C_catalyst_ V_catalyst_	Dye	C_dye_ V_dye_	C_NaBH4_ V_NaBH4_	*K_app_* or Time for Complete Reduction	Ref.
1	GSH-Ag NPs	Glutathione (GSH)	7.5×10^−9^ mol.dm^−3^ 3.9 mL	MB	0.32 mM 1 mL	0.1 mL 0.05 mol.dm^−3^	0.265 min^−1^ 18 min	[59]
2	Extracellular & Intracellular Ag NPs	Actinomycetes strains	11.3 μg/mL 1 mL	MB	5 ppm 1 mL	Photocatalytic	0.018 min^−1^ 75 min	[60]
3	LC-capped Ag NPs	Litchi chinensis (LC) fruit extract	10 mg	MB	10 ppm 50 mL	Photocatalytic	0.0335 min^−1^ 50 min	[64]
4	Biogenic Ag NPs	Piper chaba Stem Extracts	53.9 mg/L 100 µL	MB	2 ppm 2 mL	600 ppm 1.5 mL	0.17 min^−1^ 12 min	[63]
5	EPS stabilized Ag NPs	Exopolysaccharide extracted from Leuconostoc lactis KC117496	5 mg for both dyes	MO, CR	10^−5^ M 3 mL for both dyes	0.1 M 50 mL for both dyes	11.31×10^−3^ and 12.57×10^−3^ min^−1^, respectively 240 min	[61]
6	Ag NPs & GO-Ag NPs	P. quassioides bark extract for Ag NPs synthesis	0.5 mL	MB	1 µM 1.5 mL	0.01 M 1 mL	0.034 and 0.038 min^−1^, respectively 15 min	[62]
7	GC-capped Ag NPs	Green coffee extract	500 µg/mL 100 µL	MB	50 ppm 1 mL	0.1 M 1 mL	0.2867 min^−^^1^ 12 min	Present study

**Table 2:** Comparison between different biogenic silver-based nano-antioxidants and our GC-capped Ag NPs regarding the reaction and performance parameters.

Antioxidant	Source of Synthesis	IC_50_ Value	Used Free Radical	Ref.
PF@Ag NPs	Perilla frutescens (PF) leaf extract	54.52 μg/mL	DPPH	[46]
AC-Ag NPs	Ananas comosus outer peel extract	>100 µg/mL	DPPH	[66]
RT@Ag NPs	Rauvolfia tetraphylla leaves extract	82.13 µg/mL	DPPH	[67]
Ag NPs	Alternanthera sessilis Linn.	403.19 μg/mL	DPPH	[68]
IH@Ag NPs	Indigofera hisruta L. (IH) leaf extract	63.43 µg/mL	DPPH	[69]
GC capped Ag NPs	Green coffee extract	26.88 µg/mL	DPPH	Present work

## Data Availability

The data presented in this study are available on request from the corresponding author.
